# Can we respond mindfully to distressing voices? A systematic review of evidence for engagement, acceptability, effectiveness and mechanisms of change for mindfulness-based interventions for people distressed by hearing voices

**DOI:** 10.3389/fpsyg.2015.01154

**Published:** 2015-08-14

**Authors:** Clara Strauss, Neil Thomas, Mark Hayward

**Affiliations:** ^1^School of Psychology, University of SussexBrighton, UK; ^2^Sussex Partnership NHS Foundation TrustHove, UK; ^3^School of Health Sciences, Swinburne University of TechnologyMelbourne, VIC, Australia

**Keywords:** mindfulness, person-based cognitive therapy, acceptance and commitment therapy, auditory hallucinations, hearing voices, psychosis, schizophrenia

## Abstract

Adapted mindfulness-based interventions (MBIs) could be of benefit for people distressed by hearing voices. This paper presents a systematic review of studies exploring this possibility and we ask five questions: (1) Is trait mindfulness associated with reduced distress and disturbance in relation to hearing voices? (2) Are MBIs feasible for people distressed by hearing voices? (3) Are MBIs acceptable and safe for people distressed by hearing voices? (4) Are MBIs effective at reducing distress and disturbance in people distressed by hearing voices? (5) If effective, what are the mechanisms of change through which MBIs for distressing voices work? Fifteen studies were identified through a systematic search (*n* = 479). In relation to the five review questions: (1) data from cross-sectional studies showed an association between trait mindfulness and distress and disturbance in relation to hearing voices; (2) evidence from qualitative studies suggested that people distressed by hearing voices could engage meaningfully in mindfulness practice; (3) MBIs were seen as acceptable and safe; (4) there were no adequately powered RCTs allowing conclusions about effectiveness to be drawn; and (5) it was not possible to draw on robust empirical data to comment on potential mechanisms of change although findings from the qualitative studies identified three potential change processes; (i) reorientation of attention; (ii) decentring; and (iii) acceptance of voices. This review provided evidence that MBIs are engaging, acceptable, and safe. Evidence for effectiveness in reducing distress and disturbance is lacking however. We call for funding for adequately powered RCTs that will allow questions of effectiveness, maintenance of effects, mechanisms of change and moderators of outcome to be definitively addressed.

## Introduction

Auditory verbal hallucinations, (or “voices”), are a characteristic feature of psychotic conditions such as schizophrenia (American Psychiatric Association, 2013), as well as being present in a range of other conditions including borderline personality disorder (Slotema et al., 2012), post-traumatic stress disorder (Butler et al., 1996) and Parkinson's disease (Inzelberg et al., 1998) and in non-clinical populations (Daalman et al., 2011). The phenomenology of voice hearing appears similar across diagnostic and non-clinical groups (Daalman et al., 2011; Slotema et al., 2012) and the experience may or may not cause distress and disruption to functioning (Romme and Escher, 1993). For all these reasons, a symptom-based approach to understanding the voice hearing experience, associations with distress and disturbance, and therapeutic interventions is called for.

The dominant psychological therapeutic approach to working with people distressed by hearing voices has been cognitive behavioral therapy for psychosis (CBTp) which has been evaluated in a large number of randomized controlled trials and subsequent meta-analyses (Wykes et al., 2008; van der Gaag et al., 2014). The efficacy of CBTp has been questioned when looking at effects of broad measures on positive symptoms of psychosis (Jauhar et al., 2014), however, when looking at measures of hallucinations specifically, CBTp shows benefits with a moderate effect size, including in the highest quality trials (van der Gaag et al., 2014). Most research in this area has been of CBTp for people experiencing a broad range of psychotic difficulties and dedicated trials of CBT for voice hearers as a specific group are few. There is however good evidence from two randomized trials of CBT for command hallucinations that targeting beliefs about voice power reduces harmful compliance in people who have a history of complying (Trower et al., 2004; Birchwood et al., 2014). Whilst this is an important finding, the primary goal of CBTp is to reduce distress and disturbance to quality of life (Birchwood and Trower, 2006) and CBT may not reduce distress in the context of hearing voices, even when voices are the specific therapeutic target (Birchwood et al., 2014) and improvements to a wide range of quality of life variables has not been well demonstrated. An alternative approach may be warranted therefore when specifically targeting voice-related distress and disturbance.

A recent review (Thomas et al., 2014a) identified the application of mindfulness-based interventions (MBIs) as one of the most significant areas of intervention development since CBTp. Mindfulness refers to a state of consciousness that is characterized by an intentional and non-judgmental awareness of present-moment experiences (e.g., physical sensations, thoughts, sounds, voices). Rather than attempting to alter current experiences or to eliminate them from awareness, a mindful response is one that accepts what is currently present without striving to change it and without becoming absorbed in ruminating on or worrying about these experiences. Mindfulness can be considered both at the state and trait levels. Whilst the degree to which an individual is mindful might fluctuate from 1 h to the next (i.e., state mindfulness) there are also substantial individual differences between people in their tendency to be mindful (i.e., trait mindfulness) (Baer et al., 2006).

It is possible to increase trait mindfulness through MBIs (Eberth and Sedlmeier, 2012) and there is evidence that MBIs can be effective for people diagnosed with a current mental health condition. A recent meta-analysis found that MBIs relative to control conditions were effective at targeting depression symptom severity in people diagnosed with a current episode of major depression (Strauss et al., 2014) and there are a number of reasons why MBIs could also be of benefit for people experiencing distressing voices. First, MBIs are designed to increase non-judgmental acceptance of difficult experiences. Voice hearers often describe trying to resist and suppress their voices, a response which is associated with voice-related distress (Chadwick et al., 2000). The possibility that active acceptance of voices may provide a more useful alternative than seeking to eliminate them has been supported by research on coping (Farhall et al., 2007), and, amongst other approaches, is advocated by the voice-hearer led Hearing Voices Movement (Corstens et al., 2014).

Second, MBIs have been used, in particular, to target rumination and worry, transdiagnostic processes that can cause and maintain depression and anxiety (Nolen-Hoeksema et al., 2008), and a recent meta-analysis of mediation studies has shown that rumination and worry mediate the beneficial effects of MBIs on mental health outcomes (Gu et al., 2015). Rumination and worry have been reported in people with psychosis (Freeman and Garety, 1999; Thomas et al., 2014b) and, when tracking voice activity over time, rumination and worry appear to prospectively predict voice hearing episodes in day to day life (Hartley et al., 2014).

Third, voices are characteristically verbal phenomena, in which negative self-referent content can predominate (Nayani and David, 1996; McCarthy-Jones et al., 2014). Mindfulness-based interventions are thought to involve decentring from current experiences (including voices) and changing meta-cognitive beliefs about the importance and accuracy of thoughts and voice comments (Strauss and Hayward, 2013). In combination with the self-compassionate attitude fostered by MBIs (Gu et al., 2015), this may provide a means through which MBIs can buffer against the impact of negative cognition.

Fourth, whist guided approaches to talking with voices are showing promise (Corstens et al., 2012), people can also become drawn into unhelpful verbal dialogue with them (Thomas, 2015a). Mindful observation of voices provides an alternative response to talking with voices, and may disrupt an unhelpful preoccupation with internal verbal experiences in a similar way to the disruption of rumination by MBIs in depression.

There are therefore a number of good theoretical reasons why MBIs might successfully reduce voice-related distress and disturbance—through promoting acceptance, reducing rumination and worry, through increasing the ability to decenter from negative (especially self-referent) content, through changing meta-cognitive beliefs about the importance and accuracy of voice comments, and by disrupting interaction with voices. Although there are a range of theoretical reasons for offering MBIs to people distressed by hearing voices, historically caution has been advised due to concerns that lengthy mindfulness practices might exacerbate symptoms (Chadwick, 2014). Kuijpers et al. (2007) reviewed case studies and found a number of instances of meditation-induced psychotic episodes ranging from 2 days to 5 months in duration. However, the form (which may or may not have included mindfulness practice) and duration of meditation practice in these case studies is not clear and MBIs offered in clinical settings typically include relatively short mindfulness practices (< 40 min), close support by the group factiliator and limited periods of silence (Segal et al., 2002). Moreover, MBIs designed for people distressed by hearing voices are typically adapted in additional ways. This includes mindfulness practices being particularly brief (< 10 min), including guidance on attending to voices and having frequent verbal instructions with no long periods of silence (Chadwick, 2006; Dannahy et al., 2011). Despite the brief length of these practices, there is evidence that people with a mental health diagnosis can learn mindfulness (Strauss et al., 2015) and gain therapeutic benefits (Strauss et al., 2012) with these types of brief practice.

This paper presents a systematic review of the MBI for distressing voices literature with the aim of making recommendations about clinical practice and future research priorities. Whilst other reviews exist evaluating mindfulness and acceptance-based interventions for psychosis overall (Khoury et al., 2013), none, to our knowledge, have considered the specific effects of MBIs on distressing voices. Indeed, from the CBTp literature we know there are different effects on different psychotic experiences (van der Gaag et al., 2014) and a symptom-based approach that can better target core mechanisms has the potential to achieve better outcomes. There are a broad range of psychological interventions that include elements of mindfulness principles, such as Acceptance and Commitment Therapy (ACT) (Hayes et al., 1999). However, ACT is a multi-component approach of which mindfulness principles and mindfulness practice is typically only a part. Therefore, in order for this paper to explore the specific effects of mindfulness principles and practice on distressing voices we will focus on mindfulness-based interventions. We define MBIs as an intervention where the therapeutic foundation is based on mindfulness principles, where mindfulness practice is included in at least half of therapy sessions and where mindfulness home practice is encouraged. This definition allows for the inclusion studies of ACT interventions where mindfulness practice was a core part of the therapy (Shawyer et al., 2012; Bacon et al., 2014) whilst excluding ACT studies where mindfulness practice was not a core element (Bach and Hayes, 2002; Gaudiano and Herbert, 2006). In addition, as we are interested in the effects of MBIs specifically on distressing voices we are restricting this paper to a review of studies that either specifically focus on voices or that include voice-related outcomes in their data analysis.

In this systematic review we ask five questions: (1) Is there evidence that trait mindfulness is associated with reduced levels of distress and disturbance in relation to hearing voices? (2) Is it feasible to apply MBIs to people distressed by hearing voices, that is can people hearing distressing voices engage in mindfulness practice in a meaningful way and apply mindfulness to voice hearing experiences? (3) Are MBIs for distressing voices acceptable and safe, that is, are participants satisfied with the intervention, are drop-out rates low and is there evidence that distress and disturbance worsen following MBIs? (4) Are MBIs for distressing voices effective, that is, do they lead to improvements in distress and disturbance relative to a control condition and are changes sustained? (5) If effective, what are the mechanisms by which MBIs for distressing voices are having their effect? In order to address these questions we present evidence from cross-sectional studies assessing the association between trait mindfulness and voice-related constructs, qualitative studies exploring participant experiences of MBIs, single case evaluations of MBIs, uncontrolled pre-post MBI evaluations as well as RCTs of MBIs for distressing voices.

## Method

Mindfulness-based interventions were defined here as an intervention based on mindfulness principles, where mindfulness practice was included in at least half of therapy sessions and where mindfulness home practice was encouraged. Furthermore, studies with participants with psychosis, as opposed specifically for those hearing voices, were excluded unless voice-specific outcomes were reported.

Inclusion Criteria were that studies: (1) either evaluated mindfulness-based interventions (MBI) or were cross-sectional studies of mindfulness-based constructs; (2) either only included participants who had voice hearing experiences or reported findings separately for participants hearing voices; (3) were published in a peer-reviewed journal; (4) reported primary data (i.e., not reviews); (5) reported quantitative or qualitative data analysis; and (6) were available in the English language.

Titles from PsycInfo, Medline, Scopus, and Web of Science were searched on 28 February 2015 combining the terms: [(mindfulness or mindfully or acceptance or “person-based cognitive therapy”) and (psychosis or “distressing voices” or hallucination^*^ or “voice hearers” or “voice hearing” or “hearing voices” or “voice acceptance”)]. Reference sections of retrieved papers were also searched in order to identify any papers that may have been missed from the database search.

## Results

### Study characteristics

**Figure 1:**
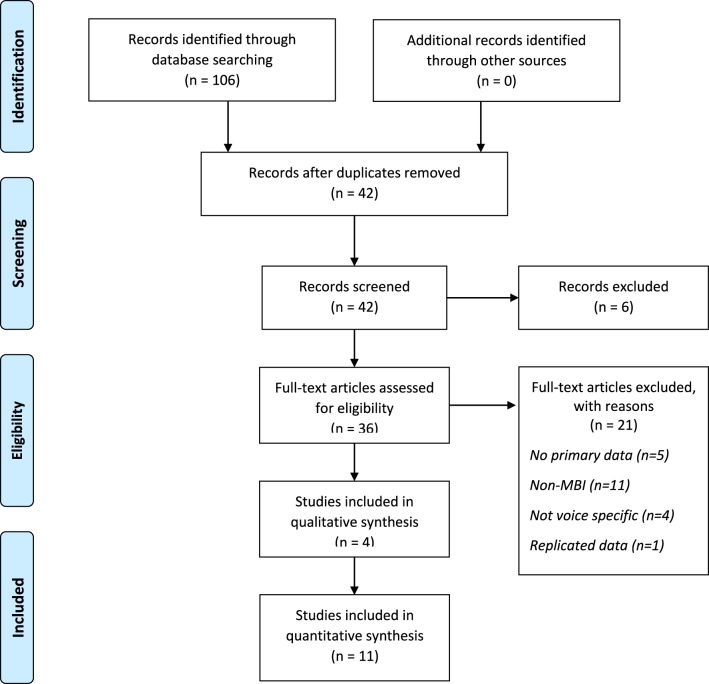
**PRISMA diagram showing study selection process**.

After removing duplicates 42 studies remained and were screened. Six studies were removed on the basis of the article title. Abstracts and full texts of the remaining 36 studies were reviewed and 21 rejected, leaving 15 studies to be included in this review. Reference sections of the final set of papers were screened and no additional studies were identified which suggests that the search strategy was sufficiently robust. Details of the search process are shown in the PRISMA diagram in Figure 1 and details of included studies are shown in Table 1.

**Table 1:** **Table of included studies**.

**References**	**Population (n)**	**Type of MBI**	**Study design**	**Key findings**
Brockman et al., 2014	40 adults meeting diagnostic criteria for schizophrenia or schizoaffective disorder and hearing voices in the past 3 months	na	Cross-sectional study reported correlations between acceptance of voices and depression, anxiety, stress, and negative affect	Acceptance of voices (as measured by the VAAS) was significantly negatively correlated with depression, anxiety, and stress (as measured by the DASS-21) and with negative affect (PANAS-negative). The size of the correlation coefficients is not reported in the paper
Chadwick et al., 2007	59 people meeting DSM-IV diagnostic criteria for schizophrenia and currently hearing voices	na	Cross-sectional study reporting correlations between measures of mindfulness, affect, and distress	Mindfulness of distressing voices (SMVQ) was negatively correlated (*r* = −0.69) with negative affect (PANAS-negative) and with self-rated distress in response to hearing voices (*r* = −0.63)
Morris et al., 2014	50 people meeting ICD-10 diagnostic criteria for a psychotic disorder or a severe depressive episode with psychotic symptoms currently hearing voices	na	Cross-sectional study reported correlations between measures of mindfulness, depression, anxiety, and behavioral and emotional resistance to voices	Mindfulness (KIMS—accept without judgment) was negatively correlated with depression (BDI-II: *r* = −0.40) and anxiety (BAI: *r* = −0.38) and with behavioral and emotional resistance to voices (BAVQ-R: *rho* = 0.42 and 0.48, respectively)
Perona-Garcelán et al., 2014	55 university students with high hallucination proneness (scoring >1 sd above mean on LSHS-R) and 28 university students with low hallucination proneness (>1 below mean on LSHS-R)	na	Cross-sectional	Participants with high hallucination proneness were less mindful (SMQ) than participants low on hallucination proneness [*t*_(81)_ = −4.56, *p* < 0.001)
Shawyer et al., 2007	43 adults diagnosed with a psychotic disorder and hearing voices during past 6 months	na	Cross-sectional study reporting correlations between measures of acceptance, depression and quality of life	Acceptance (VAAS-acceptance) was negatively correlated with depression (CDS: *rho* = −0.51) and positively correlated with quality of life (Q-LES-Q: *rho* = 0.56 and 0.35 for subjective feelings and general activities respectively)
Abba et al., 2008	16 people distressing psychosis (11 hearing voices) with voice specific effects mentioned	MBI	Qualitative using grounded theory	A three-stage process of relating differently to psychosis (not just distressing voices) was developed: centring in awareness of psychosis; allowing voices, thoughts, and images to come and go without reacting or struggle; and reclaiming power through acceptance of psychosis and the self
Bacon et al., 2014	9 with persisting positive symptoms and schizophrenia-related diagnosis	ACT including mindfulness practice in-session and encouraged for homework	Qualitative using thematic analysis	Mindfulness is one component of ACT. Amongst other components, mindfulness and acceptance were perceived as helpful, with these seen to contribute to positive changes
Goodliffe et al., 2010	18 adults receiving secondary mental health care and distressed by hearing voices. Most, but not all, had a diagnosis of schizophrenia or schizoaffective disorder	An 8-session PBCT group. A brief (< 10 min) mindfulness practice was included in sessions 5–8	Qualitative using grounded theory	One of the four derived categories was “acceptance of voices and self” However, none of the participants explicitly attributed increased acceptance to mindfulness practice and so it is possible that other elements of the therapy were responsible for a change in acceptance
May et al., 2014	10 voice hearers	12 session MBI	Qualitative using thematic analysis	Three themes were derived. The value of mindfulness emerged as a sub-theme within the “Relating to voices” theme
Newman Taylor et al., 2009	2 adults with meeting DSM-IV criteria for schizophrenia and currently hearing voices	12 weekly sessions (1 h each) of an individual MBI based on PBCT approach (Chadwick, 2006)	Case study design reporting weekly changes in self-reported distress	Both participants showed a reduction in self-reported distress (rated weekly on 0–10 scale). For participant A ratings of distress at baseline were 10/10 and 4/10 at the final follow-up and for participant B distress ratings fell from 6/10 at baseline to 0/10 at final follow-up
Chadwick et al., 2005	11 adults meeting diagnostic criteria for a psychotic disorder with 6/11 hearing voices	6 session MBI group based on PBCT approach (Chadwick, 2006)	Pre-post uncontrolled evaluation reporting pre- to post-intervention changes in psychological health	10 people provided pre- and post-therapy data. There were significant pre-post improvements on a measure of psychologist health (CORE-OM: *z* = −2.655, *p* = 0.008)
Dannahy et al., 2011	62 secondary care mental health service users distressed by hearing voices irrespective of psychiatric diagnosis	8–12 session MBI group based on PBCT approach (Chadwick, 2006; Strauss and Hayward, 2013)	Pre-post uncontrolled evaluation reporting pre- to post-intervention changes in psychological health	Using the last-observation-carried forward method to replace missing data there were significant pre-post (*MD* = 0.34, *p* < 0.001) and pre- to 1-month follow-up (*MD* = 0.38, *p* < 0.001) improvements in psychological health [CORE-OM: *F*_(2, 122)_ = 12.17, *p* < 0.001]
Chadwick et al., 2009	22 adults meeting DSM-IV diagnostic criteria for schizophrenia and hearing distressing voices for at least two years	10-session MBI group based on PBCT approach (Chadwick, 2006)	RCT of MBI group versus wait-list reporting between-group differences in psychological health and mindfulness	18 participants gave pre and post data. Differences in psychological health (CORE-OM: change score *d* = 0.56) and mindfulness (SMQ and SMVQ: change score *d* = 0.86 and 0.47, respectively) between the MBI and wait-list participants were in the hypothesized direction but not significant
Langer et al., 2010	38 university students with distressing hallucinations (distress rated at 5/10 or higher) provided complete data	8 session MBI group with each session lasting 1 h. Protocol based on MBCT protocol (Segal et al., 2002)	RCT with quasi-randomization. Active control condition involved taking part in 8 one-h video for a about social issues. Reporting between-group differences in distress and anxiety	There were no significant between-group differences in reductions distress at post-intervention, although the effect size was in the medium and in the hypothesized direction (*d* = 0.48). There was a large and significant between-group difference in improvements in anxiety (*d* = 0.88). At 16-weeks post-intervention there were non-significant effects on distress (*d* = 0.60) and a significant effect with a large effect size for anxiety (*d* = 0.91)
Shawyer et al., 2012	44 adults diagnosed with schizophrenia, schizo-affective disorder or an affective psychosis	15 individual CBT + ACT sessions including in-session mindfulness practice and home practice was encouraged	RCT comparing CBT + ACT to an active control (befriending) on a range of measures including distress and disruption in response to hearing voices	There were non-significant post-intervention between-group differences in the small to moderate range in favor of the befriending condition on measures of distress and disruption in response to voices (PSYRATS-AH distress and disruption: *d* = 0.37 and 0.52, respectively)

*BAI, Beck Anxiety Inventory (Beck et al., 1988); BAVQ-R, Beliefs about Voices Questionnaires—revised (Chadwick et al., 2000); BDI-II, (Beck et al., 1996); CDS, Calgary Depression Scale (Addington et al., 1993); CORE-OM, Clinical Outcomes in Routine Evaluation—Outcome Measure (Evans et al., 2002); DASS-21, Depression Anxiety and Stress Scales—21 item version (Henry and Crawford, 2005); KIMS, Kentucky Inventory of Mindfulness Skills (Baer et al., 2004); LSHS-R, Launay-Slade Hallucinations Scale-revised (Bentall and Slade, 1985); PANAS-negative, Positive, and Negative Affect Scale—negative affect (Crawford and Henry, 2004); PSYRATS-AH, Psychotic Symptom Rating Scales—Auditory Hallucinations (Haddock et al., 1999); Q-LES-Q, Quality of Life Enjoyment and Satisfaction Questionnaire (Endicott et al., 1993); SMQ, Southampton Mindfulness Questionnaire (Chadwick et al., 2008); SMVQ, Southampton Mindfulness of Voices Questionnaire (Chadwick et al., 2007); VAAS, Voices Acceptance and Action Scale (Shawyer et al., 2007). ACT, Acceptance and Commitment Therapy (Hayes et al., 1999); MBCT, Mindfulness-Based Cognitive Therapy (Segal et al., 2002); PBCT, Person-Based Cognitive Therapy (Chadwick, 2006)*.

There were a total of 479 participants across the 15 studies. Of these, five were cross-sectional studies assessing the association between trait mindfulness and voice-related constructs and/or global measures of distress (*n* = 247) (Chadwick et al., 2007; Shawyer et al., 2007; Brockman et al., 2014; Morris et al., 2014; Perona-Garcelán et al., 2014), four were qualitative studies of participant experiences of MBIs (*n* = 53) (Abba et al., 2008; Goodliffe et al., 2010; Bacon et al., 2014; May et al., 2014), one was a case study (*n* = 2) (Newman Taylor et al., 2009), two were uncontrolled pre-post studies (*n* = 73) (Chadwick et al., 2005; Dannahy et al., 2011), and three were RCTs (*n* = 104) (Chadwick et al., 2009; Langer et al., 2010; Shawyer et al., 2012).

### Intervention characteristics

Three forms of MBI were included across the 10 intervention studies. Seven interventional studies (Chadwick et al., 2005, 2009; Abba et al., 2008; Newman Taylor et al., 2009; Goodliffe et al., 2010; Dannahy et al., 2011; May et al., 2014) evaluated therapies based on person-based cognitive therapy (PBCT) (Chadwick, 2006). PBCT in grounded in mindfulness principles. Typically PBCT includes mindfulness practice in each therapy session and daily home practice is recommended. PBCT also draws on CBTp principles and these are integrated within a wider mindfulness-based conceptual framework (e.g., meta-cognitive belief change about the importance and accuracy of voice comments is targeted through cognitive therapy techniques and through mindfulness practice). The therapy can be delivered in groups or to individuals (Chadwick, 2006) with the number of sessions ranging from six (Chadwick et al., 2005) to 12 (May et al., 2014) or more (Newman Taylor et al., 2009). Data from two ACT trials were included (Shawyer et al., 2012; Bacon et al., 2014). In each of these in-session and between-session mindfulness practice was part of the therapy protocol. In the first of these trials (Shawyer et al., 2012) the intervention was a hybrid of ACT and CBT, combining mindfulness and other ACT methods with cognitive restructuring of beliefs about voice power. The second study (Thomas et al., 2014c) has not yet published quantitative findings however a qualitative paper has been published and was included in this review (Bacon et al., 2014). This was a trial of an 8-session pure ACT intervention for people with persisting hallucinations and/or delusions. In addition to mindfulness principles and practice ACT methods used in these trials were use of metaphors and experiential exercises to promote abandoning unproductive struggle to eliminate persisting experiences (such as voices), to defuse negative thought and voice content, to clarify personal values, and to free behavior from being dominated by avoidance and immersion in psychotic experiences (see Thomas et al. (2013) for details of the therapeutic approach). The final study (Langer et al., 2010) evaluated an adapted MBCT group (Segal et al., 2002). Group MBCT was originally designed as a relapse prevention intervention for recurrent depression. It combines a range of mindfulness practices both in-session and between-sessions with discussion about learning from mindfulness practice forming an important part of the therapy process. In this study MBCT was delivered over the usual eight sessions but sessions were half the typical length (1 h rather than the usual 2–2½h).

### Cross-sectional findings

In a student sample study, Perona-Garcelán et al. (2014), observed that participants highly prone to hallucinations [scoring 1 or more standard deviations above the mean on the Launay-Slade Hallucination Scale—revised (LSHS-R)] (Bentall and Slade, 1985) reported lower levels of mindfulness than participants who were not prone to hallucinations (scoring 1 or more standard deviations below the mean on the LSHS-R). Whilst this study did not specifically explore associations with distress and disturbance it does suggest that the tendency to experience hallucinations is associated with low levels of trait mindfulness and therefore it is plausible to speculate that trait mindfulness might protect from hallucinatory experiences.

The remaining four studies were with clinical groups experiencing voices, each finding a negative association between mindfulness constructs and distress and/or disturbance. In one study (Chadwick et al., 2007) that reported psychometric properties of the Southampton Mindfulness of Voices Questionnaire, mindfulness of voices correlated negatively with measures of negative affect, voice-related distress and resistance to voices. Similarly, in the second study, Shawyer et al. (2007) reporting the psychometric properties of the Voice Acceptance and Action Scale (VAAS), found that acceptance of voices was associated with less harmful compliance with command hallucinations, lower levels of depression, and improved quality of life. Similarly, in the third study (Brockman et al., 2014) acceptance of voices (also measured by the VAAS) was correlated with depression and anxiety. In the final study (Morris et al., 2014), a negative association was found between mindfulness (as measured by the Kentucky Inventory of Mindfulness Skills) (Baer et al., 2004) and behavioral and emotional resistance to voices as well as with depression and anxiety. Overall, all of the cross-sectional studies showed that greater mindfulness and acceptance of voices were associated with less distress (including voice-related distress, depression, and anxiety) and less disturbance (including resistance to voices, quality of life, and harmful compliance to voice commands).

These studies have used a range of measures, both of mindfulness (including general trait mindfulness and mindfulness and acceptance specifically in relation to voices) and distress (including direct measures of the impact of voices as well as general measures of depression or anxiety). Nonetheless, overall, these findings are consistent with a hypothesis that trait mindfulness can have a positive impact on distress and disturbance in the context of hearing voices. However, findings are equally consistent with a suggestion that lower levels of distress and disturbance promote greater acceptance of voices and enable people to be more mindful. That is, as data from these studies are cross sectional definitive cause-effect conclusions cannot be drawn. They do however provide a mandate for investigating the feasibility, acceptability, and effectiveness of mindfulness-based interventions and it is this evidence to which we now turn.

### MBIs: qualitative findings

A total of 53 people experiencing psychosis were interviewed about their experience of taking part in an MBI. Interview transcripts were analyzed using thematic analysis (*n* = 19) (Bacon et al., 2014; May et al., 2014) or grounded theory (*n* = 34) (Abba et al., 2008; Goodliffe et al., 2010). Many interviewees in the study by May et al. (2014) spoke of practicing mindfulness between sessions and after therapy had ended, and the majority of the interviewees in the study by Bacon et al. (2014) regarded mindfulness as a particularly useful part of the ACT intervention. However, it is striking that interviewees in the study by Goodliffe et al. (2010) offered no reflections on mindfulness practice. The authors suggested that this may be due to the very limited time made available for mindfulness practice during the therapy, with only one brief practice per session in each of the final four sessions.

Although not everyone found the experience to be helpful, and some struggled to engage with mindfulness practices, most interviewees identified a number of ways in which mindfulness had been beneficial for them. Beneficial processes included; (i) mindfulness helping to reorient attention away from voices and thereby reducing distress as “it helps me focus on something other than the voices so they don't become as distressing” (Participant 3) (Bacon et al., 2014) and (ii) mindfulness facilitating the ability to decenter and allowing oneself to “step back from your thoughts and feelings, become more aware of them” (Alison) (Abba et al., 2008) and being “able to absorb it rather than, rather than have it hit me” (Adam) (May et al., 2014). Interviewees also spoke about acquiring a different attitude toward voices—(iii) one of acceptance of voices as transient, unpleasant sensations, rather than entities that needed to be fought with and eliminated, for example, “we learnt to not put our voices out of our head, but work with them rather than try and get rid of them” (Richard) (May et al., 2014) and “voices can come and go as they please, don't get distressed, just allow it to go away” (Martin) (Abba et al., 2008).

This synthesis of findings from qualitative studies has a number of limitations. Firstly, whilst mindfulness practices were common to the experience of all interviewees, the practices were offered within the context of a range of different MBIs. Secondly, the interviewees represented only a minority of participants who received the MBIs within the trials, and it is likely that they were amongst the participants who had a more positive experience of the therapy. Finally, the study of Goodliffe et al. (2010) did not contribute to the synthesis of process themes as interviewees offered no reflections on practices. Very limited time was made available for mindfulness practice in this study, with only one brief practice per session in each of the final four sessions. This is important as it may suggest that if too little time is devoted to mindfulness practice and discussion of mindfulness principles then participants may perceive few benefits.

### MBIs: uncontrolled study findings

The two uncontrolled studies were of PBCT groups for people distressed by hearing voices (Chadwick et al., 2005; Dannahy et al., 2011). In both studies there were significant pre- to post-MBI improvements in general psychological health as measured by the CORE-OM (Clinical Outcomes in Routine Evaluation Outcome Measure) (Evans et al., 2002) with pre-post Cohen's *d* effect sizes in the moderate range (*d* = 0.53 and 0.57, respectively). The CORE-OM is a broad measure of psychological health rather than being specifically related to voices, however, Dannahy et al. (2011) additionally found significant improvements on a measure of voice distress with a large effect size (*d* = 0.75) and on a measure of voice control with a medium-large effect size (*d* = 0.62).

Whilst these findings are promising, as a control group was not included it is not possible to rule out the possibility that improvements would have occurred without therapy. What is reassuring about these findings, however, is that they do not indicate that the MBI was harmful for people distressed by hearing voices, as overall significant improvements were noted in psychological health as well as on voice-specific measures of distress and control. Moreover, 81% of participants completed therapy in the Dannahy et al. (2011) study which is an indicator of acceptability—i.e., we would assume higher rates of drop-out if the therapy was unhelpful or harmful to participants.

### MBIs: controlled study findings

Langer et al. (2010) invited 63 students who scored highly on the Revised Hallucinations Scale (RHS) (Morrison et al., 2000) and who also reported distress or anxiety in response to these experiences (at greater than 4/10) to take part in their MBI evaluation study. Thirty eight participants completed the full study (60% of those invited). Participants were allocated alternately (rather than randomly) to either the MBI condition or the control group. The MBI was an eight-session course based on mindfulness-based cognitive therapy (MBCT) (Segal et al., 2002) with 1 h sessions rather than the usual 2–2½h sessions. The active control condition involved attending eight 1 h sessions of viewing and commenting on videos of social and political relevance (e.g., religion, immigration). Data are presented for completers only and analysis showed no significant between-group differences in improvements in auditory and visual hallucinations (*d* = 0.08) or in improvements in distress, although the effect size was in the medium range (*d* = 0.48). There was however a large and significant between-group difference in improvements in anxiety (*d* = 0.88). The general pattern of findings was maintained at the 16-week post-intervention follow-up with non-significant but moderate effects on hallucinations and distress (*d* = 0.41 and 0.60) and a significant effect remaining with a large effect size for anxiety (*d* = 0.91).

The experience of hearing voices is not exclusive to those diagnosed with a psychotic condition and is perhaps more common in the non-clinical, non-help seeking population than in the clinical population (Honig et al., 1998). Despite this, non-clinical hallucinations can be associated with at least moderate levels of distress and anxiety, as demonstrated in the study by Langer et al. (2010) and there is no theoretical reason to suspect that the mechanisms through which MBIs work would be different for non-clinical and clinical groups. Effects of the MBI at improving anxiety were large relative to the control condition and sustained over a 4 month period following the intervention. Effects on distress were not significant, although, because these were moderate in size and in the hypothesized direction this may be a type II error as the study was underpowered to detect medium effects. The inclusion of an active control condition is a particular strength of this study as time and attention were controlled for. However, the 40% of participants lost to the study is a concern as it is possible that those failing to benefit were disproportionately likely to drop-out.

Chadwick et al. (2009) randomized 22 people meeting diagnostic criteria for schizophrenia and who had been distressed by hearing voices for at least 2 years to an MBI group or to a wait-list control. Participants were offered a 10 session MBI group that included mindfulness practice in session and between sessions. As this was a feasibility study, it was not powered to detect statistically significant between-group differences and all differences were non-significant. Non-significant between-group differences in improvements in psychological health (CORE-OM) were in the moderate range (*d* = 0.56), improvements in mindfulness were in the large range (*d* = 0.86) and improvements on a measure of AVHs (PSYRATS-AH) were in the small range (*d* = 0.26).

Finally, the RCT by Shawyer et al. (2012) randomized 43 people experiencing command hallucinations to either individual CBT + ACT sessions or to an active befriending condition. Again, this study was underpowered to detect anything other than large between-group effects and no between-group significant differences were found. Post-intervention between-group effect sizes on voice-related outcomes (distress and disruption) were in the small to moderate range, favoring the befriending condition (*d* = 0.37 and 0.52, respectively), which questions the benefits of the intervention condition.

In summary, to date there have only been underpowered RCTs of MBIs for distressing voices with between-group effects mostly being non-significant. The size and direction of between-group effect sizes in the studies by Langer et al. (2010) and Chadwick et al. (2009) suggest that MBIs adapted for distressing voices have promise, however, the findings from the RCT by Shawyer et al. (2012) add a note of caution to this and underline the potential risks in over interpreting effect sizes in small-scale studies. These three studies however do provide a platform for a fully powered trial of MBI for distressing voices which will help to elucidate the size of effects and ascertain the probably by which effects are due to chance.

## Discussion

This systematic review set out to answer five questions: (1) Is there evidence that trait mindfulness is associated with reduced levels of distress and disturbance in relation to hearing voices? (2) Is it feasible to apply MBIs to people distressed by hearing voices, that is can people hearing distressing voices engage in mindfulness practice in a meaningful way and apply mindfulness principles to voice hearing experiences? (3) Are MBIs for distressing voices acceptable and safe? (4) Are MBIs for distressing voices effective? (5) If effective, what are the mechanisms by which MBIs for distressing voices are having their effect?

Fifteen studies meeting inclusion criteria were included in the review. In relation to the five review questions: (1) data from cross-sectional studies were consistent in showing an association between trait mindfulness and distress and disturbance in relation to hearing voices; (2) evidence from qualitative studies suggested that people distressed by hearing voices could engage meaningfully in mindfulness practice, including outside of therapy sessions; (3) MBIs were seen as acceptable (high rates of satisfaction and low drop-out rates) and safe (with pre-post MBI improvements on measures of distress and disturbance); (4) there were no adequately powered RCTs allowing us to draw conclusions about effectiveness in reducing distress and disturbance; and (5) given the lack of effectiveness studies it was not possible to draw on robust empirical data to comment on potential mechanisms of change although findings from the qualitative studies identified three potential change processes; (i) reorientation of attention; (ii) decentring; and (iii) acceptance of voices.

In relation to question 1 the review of cross-sectional studies found consistent evidence that trait mindfulness was associated with reduced hallucination proneness and, more importantly from a therapeutic perspective, that mindfulness is associated with reduced levels of distress (including voice-related distress as well as depression and anxiety) and reduced disturbance (including quality of life and harmful compliance to command hallucinations). These findings corroborate findings in the broader mindfulness literature that trait mindfulness is associated with a wide range of mental health and well-being variables (Brown and Ryan, 2003). Whilst the direction of the relationship between mindfulness, distress, and disturbance cannot be determined from such cross-sectional studies the findings are consistent with (though not proof of) the suggestion that mindfulness might lead to reduced distress and disturbance in response to hearing voices. We can therefore further speculate that training people in mindfulness through MBIs might help to alleviate voice-related distress and disturbance.

The review of qualitative studies (question 2) suggest that at least some people experiencing distressing voices can engage meaningfully in mindfulness practices in MBIs and this finding is important as it has been suggested that distressing voices might be a barrier to meaningful engagement (Kuijpers et al., 2007). Contrary to this suggestion, participants reported practicing mindfulness outside of therapy sessions—at home and also after the therapy had ended—suggesting a willingness to practice beyond the confines of a therapy group. In addition to formal mindfulness practices, participants reported bringing mindfulness to voice hearing experiences in daily life. A caveat to these findings however is that participants willing to take part in qualitative interviews may not have been representative of the wider study sample and they may have been disproportionately likely to have had a positive experience of the therapy. This means that it is possible that some participants struggled to engage with mindfulness practice but that their perspectives were not included in the qualitative analyses. Future qualitative research should actively attempt to recruit participants who have found engagement difficult (e.g., interviewing those people dropping out from MBI groups) in order to explore this possibility further.

Mindfulness-based interventions for distressing voices appear to be acceptable to participants (question 3) both as ratings of satisfaction were high (Bacon et al., 2014) but also because drop-out rates were low (Dannahy et al., 2011), a reasonable proxy for acceptability. They also appear to be safe, at least on a group level, as pre- to post-MBI improvements in distress and disturbance were found in the quantitative evaluation studies. This suggests at the very least that the MBIs under investigation were not leading to (group-level) worsening of difficulties in terms of distress and disturbance, although some evidence was found for increases in voice activity following mindfulness practice (Bacon et al., 2014). The lack of evidence for exacerbating distress along with low rates of therapy drop-out and high satisfaction ratings are of interest and clinical relevance as historically people experiencing active symptoms of psychosis have often been excluded from MBIs due to concerns about detrimental effects (Chadwick, 2014). It is important to note however that all the MBIs evaluated in this review included adapted, brief mindfulness practices. Mindfulness-based interventions such as MBCT include much longer mindfulness practices (up to 40 min) and our findings do not allow us to comment on the acceptability or safety of these longer practices. As such we would recommend the use of brief practices, with the most common form of practice used in the studies in this review taken from Chadwick (2006).

The question of effectiveness in improving distress and disturbance (question 4) is still outstanding due in part to only having findings from underpowered studies to draw on. In the current review one trial with students distressed by hallucinatory experiences (Langer et al., 2010) did find improvements in anxiety following an MBI relative to a control condition and improvements were sustained for 16 weeks following therapy. However, the two RCTs with clinical samples were underpowered to detect anything other than large effect sizes and the lack of significant between-group differences on measures of distress and disturbance are therefore difficult to interpret. Whilst the pilot RCT by Chadwick et al. (2009) found non-significant effects in favor MBI in comparison to the wait-list control group, the larger RCT by Shawyer et al. (2012) found non-significant between-group effects on measures of voice-related distress and disruption in favor of the befriending control condition. This latter finding is important as it highlights the need for an adequately powered RCT that is able to answer questions of effectiveness definitively. Therefore, as yet we do not know if MBIs for distressing voices are effective at targeting voice-related distress and disturbance and as such we would advocate caution in routinely offering these groups in clinical settings.

The lack of certainty about effectiveness, coupled with the multi-component nature of many of the interventions under review, means that it is also somewhat premature to answer questions about mechanisms of change (question 5). Findings from the four qualitative studies shed some light on possible therapeutic processes however. In particular, becoming more mindful was seen by participants to enhance the ability to: (i) reorient attention; (ii) cultivate the ability to decenter from difficult thoughts and voices; and (iii) accept voices. These processes are in line with three of the five proposed mechanisms of change outlined in the introduction. We suggested that MBIs for distressing voices could potentially be of benefit through reducing interaction with voices, enabling decentring from voice hearing experiences and increasing acceptance of voices. Current qualitative findings are in line with these suggestions but further scrutiny of effectiveness and potential mechanisms of change is now needed.

### Limitations

This review is limited in its ability to answer all of the five posed questions as this is an area of research in its early stages. Whilst the evidence to date allows us to suggest that research investigating MBIs for distressing voices is warranted and that MBIs for distressing voices can be meaningful engaged with, acceptable and safe we do not yet know if these interventions lead to improvements in distress and disturbance, if improvements can be sustained over time or what the precise mechanisms of change are that underlie improvements, although findings from the qualitative studies begin to elucidate what these mechanisms might be.

A significant limitation is a lack of adequately powered RCTs. This makes it difficult to distinguish between a genuine lack of effectiveness on the one hand and genuine effects that have not been detected on the other (i.e., type II errors). Now that we can more confidently answer the first three of our posed questions future research can focus on addressing questions of effectiveness (question 4) in definitive RCTs. Effectiveness questions should not just focus on short-term outcomes but also on maintenance of any changes, as if effects are quickly lost following the end of the therapy it is questionable if it is a good use of scarce therapeutic resources to invest in MBIs for distressing voices.

A further limitation is that even if MBIs for distressing voices are effective we do not know the mechanisms by which they might be having their effects (question 5) and most studies in this review failed to measure the broad range of potential mechanisms that might be at play. As noted earlier, theoretically we would expect MBIs for distressing voices to work through a number of mechanisms including enhancing acceptance, reducing rumination and worry, increasing decentring (especially in relation to negative self-referent voice content), increasing self-compassion and disengaging from interacting with voices. Whilst definitive RCTs could better address questions of effectiveness these would be further enhanced by including measures of these mechanisms during the MBI process and using mediation analyses to investigate which, if any, are important mechanisms of therapeutic change.

A particular limitation in this review and in the field more broadly is the lack of consensus on primary therapeutic targets and on agreeing on reliable and valid measures of these targets. We argue along with others (Birchwood and Trower, 2006) that the primary aim of psychological therapies for distressing voices is to reduce distress and disturbance. However, precisely what is meant by this is not well-established. By reducing distress do we mean specifically in relation to hearing voices, and so we would want valid and reliable measures of voice-related distress, or do we mean distress more globally, beyond voices, in which case we might want to focus on valid and reliable measures of depression and anxiety? By reducing disturbance do we mean specifically in relation to voices, such as compliance with harmful command hallucinations, or do we mean globally? If the latter, what do we mean by and how do we measure disturbance? Might this be through measuring recovery, time use, life satisfaction or quality of life? These are clearly still open questions without agreement and we would do well to reach a consensus amongst researchers in the field before moving forward with large research trials.

Finally, many of the intervention studies in this review included non-mindfulness components in their interventions through the inclusion of elements from CBTp (Goodliffe et al., 2010; Dannahy et al., 2011; Shawyer et al., 2012; May et al., 2014) and values-directed behavior change elements from ACT (Shawyer et al., 2012; Bacon et al., 2014). Going forward and needing a clearer understanding about the effectiveness of MBIs for distressing voices and their mechanisms of change it would be worthwhile exploring the specific-effects of learning mindfulness independent of effects of other potentially therapeutic elements.

### Future research

Questions for future research arise from the limitations noted above. A research priority is to reach a consensus in the field on the primary therapeutic targets for MBIs for distressing voices specifically but also ideally for psychological therapies for distressing voices more broadly. If this could be achieved a next step would be to agree on a core set of valid and reliable measures and this could involve a process of developing and testing new measures. Challenges should be noted given limitations in metacognitive ability in this client group which might make reliable self-report difficult to achieve using standard self-report tools (Farhall et al., 2013). New technologies including neuroimaging methods applied to brain regions involved in voice-hearing and inner verbalization, and ecological momentary assessment of preoccupation with voices may provide useful complements to these traditional self-report measures (Thomas, 2015b).

Another priority for future research is to conduct adequately powered RCTs of MBIs that conform to CONSORT criteria, that include long-term follow-up, that include control conditions that themselves do not contain active elements of the therapy under investigation and that include measures of the proposed mechanisms outlined above. These studies could provide answers to the questions 4 and 5 that we posed but were unable to adequately answer and would help to elucidate mechanisms of change (if any). Elucidating mechanisms of change would mean that therapy protocols for MBIs for distressing voices could be further refined to better target the most important of the mechanisms and thereby potentially lead to improved outcomes.

Last but not least, we perhaps need to pay better attention to what works for whom across the psychological therapies for distressing voices field. It may well be the case that “one size does not fit all” and that different therapeutic approaches (e.g., CBTp, ACT, MBI) benefit different groups of people. For instance, neuropsychological factors such as attention do not seem to be a barrier to CBTp (Premkumar et al., 2011) but may impede the ability to engage with and benefit from MBIs given the emphasis on sustained attention to present moment experience. Whilst expensive to conduct, questions of moderation of effect could be included in future RCTs comparing different therapeutic approaches with a view to elucidating what works for whom or indeed whether MBIs can be effective for people with a broad range of presentations (see Hayward et al., this volume for further discussion of the “what works for whom” question).

## Conclusions

In this systematic review we set out to answer five questions. We have shown that mindfulness-based interventions adapted for distressing voices are warranted (as trait mindfulness was associated with distress and disturbance in people hearing voices), engaging, acceptable, and safe. Potential mechanisms of change have been highlighted through qualitative studies including reorientation of attention, decentring from voices and acceptance of voices. There are however insufficient randomized controlled trials to date to allow us to answer questions about effectiveness—we cannot say whether or not MBIs for people distressed by hearing voices are effective at targeting distress and disturbance either in the short or longer term. Future research therefore needs to focus on adequately powered RCTs with long-term follow-up and measurement of proposed MBI mechanisms and moderators. Before this research is taken forward we suggest that an attempt is made to reach consensus in the psychological therapies for distressing voices field as to primary therapeutic targets and measurement tools for these targets. If this could be achieved it will allow us to better integrate findings across studies with a view to providing definitive answers to the questions about the effectiveness and mechanisms of change of MBIs for distressing voices and thereby offering people distressed by hearing voices an informed choice about therapeutic approaches.

### Conflict of interest statement

The authors declare that the research was conducted in the absence of any commercial or financial relationships that could be construed as a potential conflict of interest.
